# Reasons for Termination of Treadmill and Cycle Ergometer Exercise Tests in Patients After Myocardial Infarction: The Role of Clinical Profile and Exercise Capacity

**DOI:** 10.3390/jcm15145638

**Published:** 2026-07-18

**Authors:** Beata Czechowska, Jacek Chrzczanowicz, Rafał Gawor, Joanna Kostka

**Affiliations:** 1 Department of Methodology of Teaching Movement, Medical University of Lodz, Pl. Hallera 1, 90-647 Lodz, Poland; beata.czechowska@umed.lodz.pl; 2 Cardiac Rehabilitation Centre, Copernicus Memorial Hospital, Popioly 40, 93-438 Lodz, Poland; jacekchrz@interia.pl (J.C.); gawor@onet.eu (R.G.); 3 Department of Physioprophylaxis, Medical University of Lodz, Pl. Hallera 1, 90-647 Lodz, Poland

**Keywords:** exercise testing, treadmill, cycle ergometer, cardiac rehabilitation, myocardial infraction, PCI (percutaneous coronary intervention)

## Abstract

**Background**: Exercise testing is routinely performed before initiation of phase II cardiac rehabilitation to assess exercise capacity and determine safe training intensity. However, exercise tests may be terminated before reaching the target heart rate due to various cardiovascular and non-cardiovascular factors. The aim of this study was to analyze the reasons for terminating exercise tests performed on a treadmill and a cycle ergometer, with particular emphasis on the impact of the patient’s clinical profile and exercise capacity. **Methods**: A retrospective analysis was conducted on 336 patients after myocardial infarction (74 women and 262 men) referred to inpatient phase II cardiac rehabilitation between 2019 and 2022. Patients were divided into two matched groups according to the type of exercise test performed: treadmill and cycle ergometer. Reasons for test termination, exercise performance parameters, and demographic and clinical characteristics were analyzed. **Results**: The most common reasons for exercise test termination were fatigue (55.1%) and achievement of the target heart rate (37.2%), with no significant differences between testing modalities. Lower limb pain was significantly more frequent during cycle ergometer testing compared with treadmill testing (16.1% vs. 7.1%; *p* = 0.011). Patients tested on the treadmill achieved significantly higher exercise capacity (7.19 vs. 5.28 METs (metabolic equivalents); *p* < 0.001), higher peak heart rate, and higher rate-pressure product. Multiple regression analysis showed that the association between exercise modality and lower limb pain was not independent after adjustment for clinical and demographic variables, indicating that the observed differences were largely explained by patient clinical characteristics. **Conclusions**: The most common reasons for exercise test termination were patient fatigue and attainment of the target heart rate, confirming the safety of submaximal exercise testing in post-myocardial infarction patients. Lower limb pain was more frequently observed during cycle ergometer testing in unadjusted analysis; however, this association was not independent of clinical variables, indicating that it is primarily related to the patient’s clinical profile rather than the exercise modality. Exercise testing should therefore be individualized according to clinical status, functional capacity, and rehabilitation goals.

## 1. Introduction

The primary tools used in both cardiological diagnosis and the assessment of eligibility for cardiac rehabilitation are exercise tests performed on a treadmill or stationary bicycle. They enable the functional assessment of the cardiovascular and respiratory systems, analysis of the body’s response to controlled physical exertion, and identification of factors limiting exercise tolerance [1,2,3]. They also enable the detection of symptoms of myocardial ischemia, cardiac arrhythmias, and abnormal hemodynamic responses to physical exertion [3,4].

According to the current European Society of Cardiology (ESC) Guidelines on Sports Cardiology and Exercise in Patients with Cardiovascular Disease (2020) [5] and the European Association of Preventive Cardiology (EAPC) recommendations (2021) [1,2], exercise testing before the initiation of cardiac rehabilitation is recommended to assess functional capacity, identify exercise-limiting factors, and prescribe individualized and safe exercise training intensity.

Cardiopulmonary exercise testing (CPET) is considered the gold standard for the objective assessment of exercise capacity because it provides direct measurement of oxygen uptake (VO_2_peak) and a comprehensive evaluation of cardiovascular, respiratory, and metabolic responses to exercise. However, due to its limited availability, higher cost, and the need for specialized equipment, CPET is not routinely performed in many cardiac rehabilitation centers. Consequently, conventional exercise testing on a treadmill or cycle ergometer remains the most widely used method for functional assessment and exercise prescription in everyday clinical practice.

As part of the screening process for the second stage of outpatient cardiac rehabilitation, submaximal exercise tests are most commonly performed. Their purpose is to reach a predetermined heart rate limit, which allows for the assessment of exercise capacity and the customization of the rehabilitation program [2,3]. In clinical practice, however, reaching the target exercise intensity is not always possible. The test may be terminated prematurely due to the onset of clinical symptoms, such as chest pain, shortness of breath, dizziness, or excessive fatigue, as well as due to changes in the electrocardiogram, cardiac arrhythmias, or an abnormal blood pressure response [3,4]. Non-cardiac factors may also cause the test to be interrupted, including orthopedic limitations, musculoskeletal pain, and low exercise tolerance [4].

Treadmills and bicycle ergometers are the most commonly used devices for conducting exercise tests, but they differ in their exercise characteristics. During exercise on a treadmill, a greater amount of muscle mass is engaged, which is usually associated with higher values of maximum oxygen uptake (VO_2_max), a higher heart rate, and greater exercise capacity than during tests performed on a bicycle ergometer [6]. In contrast, the bicycle ergometer is often preferred for individuals with balance disorders, orthopedic limitations, or an increased risk of falling [2,6]. These differences may affect not only the exercise parameters obtained but also the reasons for terminating the test.

The course and outcome of an exercise test are also influenced by the patient’s individual characteristics. With age, there is a gradual decline in aerobic capacity and a higher prevalence of conditions that limit physical activity [7]. Gender may also be significant, as women and men differ in body composition, muscle mass, hemodynamic response to exercise, and subjective perception of fatigue [8,9]. These factors can influence both exercise tolerance and the type of symptoms leading to the termination of the exercise test. Moreover, some patients after myocardial infarction present with mildly reduced left ventricular ejection fraction (HFmrEF), in whom comprehensive cardiac rehabilitation combined with guideline-directed pharmacological therapy has been shown to improve functional capacity, quality of life, and clinical outcomes. Recent evidence highlights that individualized exercise prescription based on functional assessment is a key component of the management of this patient population, emphasizing the importance of selecting the most appropriate exercise testing modality [10].

Although differences in exercise performance parameters, such as metabolic equivalent (MET) and heart rate (HR), achieved during treadmill and cycle ergometer tests are well documented in the literature [6], most studies to date have focused primarily on assessing prognostic and exercise performance parameters. However, available data on the direct causes of exercise test termination in patients after myocardial infarction treated with percutaneous coronary intervention (PCI) remain limited, particularly regarding the relationship between these causes, the exercise modality used, and the patient’s clinical profile [1]. Better understanding of these relationships may contribute to optimizing exercise testing strategies and further individualizing cardiac rehabilitation programs.

The aim of this study was to analyze the reasons for terminating exercise tests performed on a treadmill and a bicycle ergometer, with particular emphasis on the impact of the patient’s clinical profile and exercise capacity.

## 2. Materials and Methods

### 2.1. Study Design and Participants

This study was conducted using the medical records of patients treated at the Early Cardiac Rehabilitation Center in Łódź, Poland. The analysis included exercise test results obtained before the initiation of phase II inpatient cardiac rehabilitation. Patients with a history of myocardial infarction were eligible for inclusion, including both ST-segment elevation myocardial infarction (STEMI) and non-ST-segment elevation myocardial infarction (NSTEMI). All patients enrolled in the study underwent successful revascularization via PCI before beginning rehabilitation.

**Figure 1:**
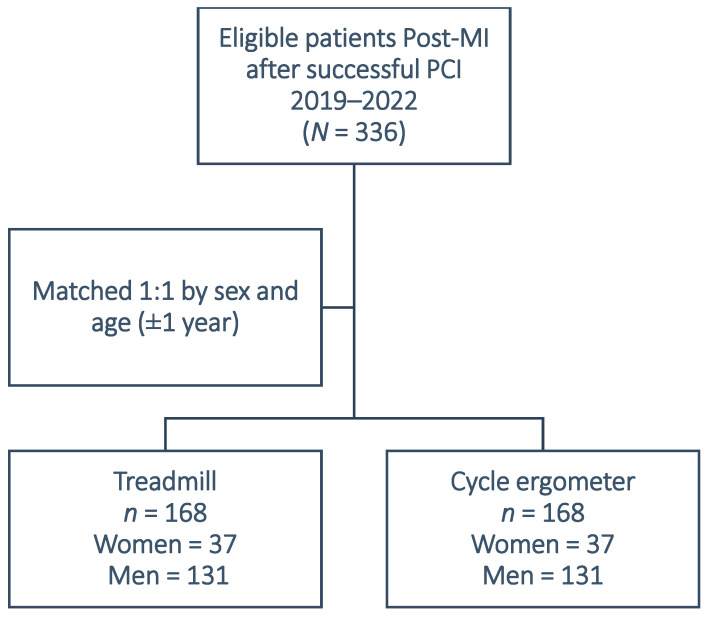
Flow chart of patient selection and allocation to treadmill and cycle ergometer exercise testing (authors’ own elaboration).

A total of 336 patients admitted to the rehabilitation center between 2019 and 2022 were included in the study, comprising 74 women and 262 men. Two groups of patients were analyzed: those who underwent treadmill exercise testing (*n* = 168) and those who performed cycle ergometer exercise testing (*n* = 168). Patients were assigned to either treadmill or cycle ergometer testing by a physician according to routine clinical practice. The choice of testing modality resulted from organizational factors and equipment availability within the rehabilitation center while maintaining patient safety and considering contraindications to specific exercise testing methods. To ensure comparability between groups, participants were matched 1:1 according to sex and age (±1 year). Consequently, both the treadmill and cycle ergometer groups consisted of an equal number of women (*n* = 37) and men (*n* = 131), with no significant differences in mean age (Figure 1).

The inclusion criteria were age ≥18 years, referral for inpatient cardiac rehabilitation, complete medical records, and an available exercise test result. Exclusion criteria included age <18 years, absence of an exercise test, or incomplete medical documentation.

The study was approved by the Director of the rehabilitation center and received a favorable opinion from the Bioethics Committee of the Medical University of Łódź (approval no. RNN/17/24/KE, issued on 9 January 2024).

### 2.2. Exercise Testing Procedures

Exercise tests were performed according to a standardized protocol routinely used at the Early Cardiac Rehabilitation Center in Łódź. All examinations were conducted by a qualified cardiac rehabilitation team consisting of a physician and a physiotherapist.

Before each test, participants underwent a clinical assessment, resting electrocardiographic (ECG) evaluation, and measurements of blood pressure and heart rate. Continuous ECG and heart rate monitoring were maintained throughout the examination. Blood pressure was measured at rest, immediately before exercise, and at the end of each exercise stage. Following completion of the test, patients remained under observation until hemodynamic parameters stabilized and any ECG abnormalities resolved.

Exercise testing was performed using a Cardiovit AT-2 Plus system (SCHILLER AG, Baar, Switzerland) integrated with a TMX 425 treadmill (Full Vision Inc., Newton, KS, USA) and an ErgoSelect 100P cycle ergometer (ergoline GmbH, Bitz, Germany). Before testing, all participants received standardized instructions regarding the procedure and were informed to report symptoms requiring test termination, including chest pain, dizziness, dyspnea, nausea, or excessive fatigue [5].

### 2.3. Treadmill Exercise Testing

Treadmill exercise testing was performed according to the Bruce protocol [11]. Exercise intensity was progressively increased every 3 min by simultaneously increasing treadmill speed and incline. Exercise intensity was expressed in metabolic equivalents (METs).

### 2.4. Cycle Ergometer Exercise Testing

Cycle ergometer exercise testing was performed according to a RAMP protocol (Continuous Incremental Workload Protocol) [12]. The test began with a 2–3 min warm-up period without resistance. Following the warm-up, the workload was increased continuously and linearly at a rate of approximately 8.3 Watts per minute (corresponding to an average increment of 25 W every 3 min) until exercise termination criteria were met. Exercise intensity was expressed in watts (W) and metabolic equivalents (METs).

Both treadmill and cycle ergometer tests were followed by a seated recovery period during which ECG recordings, blood pressure, and heart rate were continuously monitored until values returned close to resting levels.

### 2.5. Exercise Test Termination Criteria

All participants underwent submaximal exercise testing. The test was terminated when 85% of the predicted maximal heart rate was achieved or when clinical indications for discontinuation occurred [11,12]. Since all patients were receiving β-blocker therapy, the target heart rate limit was reduced by an additional 10% for the entire study group.

Based on exercise test reports, the following reasons for test termination were identified:

- Patient exhaustion (general fatigue);
- Achievement of the target heart rate;
- Achievement of the blood pressure limit;
- Cardiac arrhythmias;
- ECG abnormalities;
- Lower-limb pain or discomfort (non-specific muscular or peripheral pain);
- Other reasons, including chest pain, dyspnea, and dizziness.

Exceeding the blood pressure limit was defined as a systolic blood pressure >250 mmHg, a diastolic blood pressure >115 mmHg, or a decrease in systolic blood pressure >10 mmHg during exercise [11,12,13].

Perceived exertion was assessed using the Borg Rating of Perceived Exertion Scale ranging from 6 to 20 points, where 6 indicated no exertion and 20 indicated maximal exertion [5,14].

### 2.6. Variables and Outcome Measures

The primary outcome variable was the reason for exercise test termination. Additionally, the influence of age and sex on the frequency of specific causes of test discontinuation was evaluated.

The following demographic and clinical variables were obtained from medical records:

- Age;
- Sex;
- Body weight;
- Body height;
- Body mass index (BMI);
- Comorbidities;
- Number of medications used;
- Left ventricular ejection fraction (LVEF).

The total number of prescribed medications was used as an indirect indicator of overall clinical burden and multimorbidity rather than as a measure of specific pharmacological treatment.

Selected exercise test parameters were also analyzed, including resting heart rate (HRrest), peak heart rate (HRpeak), heart rate recovery (HRR), systolic blood pressure (SBP) and diastolic blood pressure (DBP), metabolic equivalents (METs), double product (DP), double product reserve (DP reserve), and perceived exertion.

- Heart rate recovery was calculated according to the following formula [1,15]:

HRR = HRmax − HR1min

Formulas for determining METs for a treadmill and a bicycle ergometer (V = velocity (treadmill speed); G = grade (treadmill incline)) [16]:

- Treadmill (V < 8 km/h): MET = (V × 1.675 + 0.3015 × V × G + 3.5)/3.5;
- Treadmill (V ≥ 8 km/h): MET = (V × 3.35 + 0.15075 × V × G + 3.5)/3.5;
- Cycle ergometer: MET = (12 × workload (W) + 3.5 × body mass (kg))/(body mass (kg) × 3.5);
- Double product (DP), an indirect indicator of myocardial oxygen demand, was calculated for both resting and peak exercise conditions according to the formula [17,18,19]:
  DP = (SBP × HR)/100;
- Double product reserve (DP reserve), reflecting the increase in myocardial oxygen demand during exercise relative to rest, was calculated as follows [17,20]:
  DP reserve = DPpeak/DPrest.

Perceived exertion was assessed using the Borg 6–20 scale [5,14].

### 2.7. Statistical Analysis

Statistical analyses were performed using Statistica version 13.3 (TIBCO Software Inc., San Ramon, CA, USA). Microsoft Excel 2019 was used for preliminary data collection and data management.

Continuous variables were described using descriptive statistics, including mean, standard deviation (SD), minimum value, and maximum value. Data normality was assessed using the Shapiro–Wilk test. Depending on the distribution of variables, between-group comparisons were performed using Student’s *t*-test for normally distributed variables or the Mann–Whitney U test for non-normally distributed variables. Categorical variables were analyzed using Pearson’s chi-square test or Fisher’s exact test, as appropriate.

To evaluate the influence of selected factors (sex, age, BMI, and exercise testing modality) on the occurrence of lower limb pain, but to evaluate the independence of the exercise modality’s effect after adjusting for key clinical and demographic variables. A *p*-value < 0.05 was considered statistically significant.

## 3. Results

Table 1 presents a detailed description of the study group (*N* = 336), broken down by type of exercise test (treadmill vs. bicycle) and gender. The results indicate no significant differences in the age of the participants; however, significant differences were observed in BMI (*p* = 0.012) and left ventricular ejection fraction (LVEF), which was higher in those who performed the test on a treadmill (*p* = 0.006) (Table 1).

In both groups, overweight was the most prevalent BMI category, affecting 49.4% of patients who underwent treadmill exercise testing and 46.4% of those who performed cycle ergometer exercise testing. Normal body weight was observed in 17.9% and 28.0% of participants, respectively, whereas obesity was present in 32.7% and 25.6% of patients. No statistically significant differences were found in the distribution of BMI categories between the groups (*p* = 0.185).

All patients included in the study had undergone successful percutaneous coronary intervention (PCI) following their myocardial infarction prior to the initiation of the cardiac rehabilitation program.

Analysis of the prevalence of individual comorbidities revealed no statistically significant differences between patients who underwent treadmill exercise testing and those who performed cycle ergometer exercise testing, either in the overall study population or after stratification by sex (*p* > 0.05).

In the overall study population, arterial hypertension was the most prevalent comorbidity, occurring in 73.81% of patients who underwent treadmill exercise testing and 79.17% of those who performed cycle ergometer exercise testing (*p* = 0.207). Diabetes mellitus was present in 20.24% and 27.38% of patients, respectively (*p* = 0.124), whereas osteoarthritis was diagnosed in 19.05% of patients in the treadmill group and 21.43% of patients in the cycle ergometer group (*p* = 0.587). Other conditions, including rheumatic diseases, chronic respiratory, gastrointestinal, and kidney diseases, benign prostatic hyperplasia, thyroid disorders, cancer, peripheral arterial disease, and lower-limb varicose veins, were observed less frequently.

Among women, arterial hypertension was the most common comorbidity, affecting 75.68% of patients in the treadmill group and 78.38% of patients in the cycle ergometer group (*p* = 0.782). Diabetes mellitus was the second most prevalent condition, occurring in 13.51% and 21.62% of women, respectively (*p* = 0.271).

Similarly, among men, arterial hypertension was the predominant comorbidity, present in 73.28% of patients in the treadmill group and 79.39% of those in the cycle ergometer group (*p* = 0.200). The next most frequently observed conditions were diabetes mellitus (22.14% vs. 29.01%; *p* = 0.203) and osteoarthritis (19.85% vs. 20.61%; *p* = 0.878).

However an analysis of the total burden of disease revealed that patients who underwent the test on a stationary bike had a mean number of comorbidities (3.33 ± 1.42 vs. 2.80 ± 1.51; *p* < 0.001) and were taking more medications (8.05 ± 2,10 vs. 7.47 ± 1.82; *p* = 0.001) compared to the treadmill group (Table 1).

In the study group, patients who underwent a treadmill test achieved significantly higher MET values compared to those who underwent a cycle ergometer test (7.19 vs. 5.28; *p* < 0.001). For heart rate (HR) and the double product (DP), the analysis also showed significantly higher values in the group of patients who underwent the test on a treadmill, in both women and men (Table 2).

The most common reasons for discontinuing the exercise test in the study group were fatigue (55%) and reaching the heart rate limit (37%), with no significant differences between the groups. Pain in the lower extremities was significantly more common as a reason for discontinuing the exercise test on a stationary bike (16%) than on a treadmill (7%), with a *p*-value of 0.011 (Table 3).

In the group of women, lower limb pain occurred more frequently among those cycling (21.62%) than among those on the treadmill (10.81%); however, no statistically significant differences were observed in the reasons for terminating the exercise test depending on the equipment used (*p* > 0.05) (Table 4).

In the group of men, the analysis showed that lower limb pain was a significantly more common cause of premature termination of the test on the bicycle ergometer (14.5%) compared to the treadmill (6.11%), which was statistically significant (*p* = 0.025) (Table 5).

A multiple regression analysis was performed with test termination due to lower limb pain as the dependent variable. The primary model (Model 1) included exercise modality (treadmill vs. cycle ergometer), sex, age, and BMI as independent variables. Within this model, exercise modality was the only variable significantly associated with test termination due to lower limb pain (β = −0.145, *p* = 0.008). However, the model as a whole reached only borderline statistical significance (F(4,331) = 2.30, *p* = 0.059) and explained a small proportion of the variance (R^2^ = 0.027). Therefore, these findings should be interpreted with caution and regarded primarily as an exploratory analysis rather than evidence of a clinically meaningful predictive relationship.

After expanding the model to include additional clinical variables (left ventricular ejection fraction [LVEF], exercise capacity expressed as METs, and the number of comorbidities), exercise modality was no longer significantly associated with test termination due to lower limb pain (*p* > 0.05). The expanded model explained only a small proportion of the variance (F(7,321) = 1.71, *p* = 0.105; R^2^ = 0.036), indicating limited explanatory power. Therefore, these findings should be interpreted cautiously. Nevertheless, the loss of statistical significance for exercise modality after adjustment suggests that the unadjusted association may have been influenced by differences in the clinical characteristics of patients assigned to each testing modality rather than by the exercise device itself.

## 4. Discussion

This study investigated the reasons for termination of exercise tests performed on a treadmill and a cycle ergometer in patients following myocardial infarction undergoing phase II cardiac rehabilitation refers to the early supervised outpatient rehabilitation program for clinically stable patients following myocardial infraction.

The predominant reason for test discontinuation was patient fatigue, which is consistent with previous reports in cardiac populations, where subjective exercise intolerance represents a primary determinant of test cessation [3,4]. The second most frequent cause was attainment of the target heart rate, reflecting the use of a submaximal protocol applied for safety reasons during rehabilitation qualification. The low incidence of termination due to electrocardiographic abnormalities, arrhythmias, or abnormal blood pressure responses further supports the safety profile of both exercise modalities, in line with large-scale observational data reporting a very low rate of adverse events during exercise testing [21,22].

Although age, sex, and BMI are known determinants of exercise performance, influencing aerobic capacity, maximal heart rate, and symptom perception [7,8,9], no significant associations were observed in relation to lower limb pain as a termination criterion in the present cohort.

A higher frequency of lower limb pain was initially observed during cycle ergometer testing. However, this association was not confirmed in multivariable analysis after adjustment for key clinical variables, including exercise capacity (MET), left ventricular ejection fraction, and comorbidity burden. These findings suggest that the observed differences are more closely related to patient clinical characteristics than to the testing modality itself.

Patients referred for cycle ergometer testing demonstrated a higher comorbidity burden, lower left ventricular ejection fraction, and reduced functional capacity. In this context, peripheral limitations related to muscular fatigue and discomfort may emerge earlier than centrally mediated cardiovascular limitations. Additionally, biomechanical differences between modalities may contribute to this phenomenon. Cycle ergometry imposes a sustained, localized workload predominantly on the quadriceps and gluteal muscles, whereas treadmill walking involves a more distributed muscle activation pattern and better reflects everyday functional movement. Consequently, peripheral fatigue may occur earlier during cycling, potentially leading to earlier test termination.

The study population had a mean LVEF of 49–52%, corresponding to heart failure with mildly reduced ejection fraction (HFmrEF). This represents a clinically relevant and heterogeneous subgroup of post-myocardial infarction patients, characterized by variable functional capacity and comorbidity burden, requiring individualized exercise prescription and close clinical monitoring during rehabilitation [10].

Despite higher external workload parameters (MET, peak heart rate, and rate-pressure product) achieved during treadmill testing, perceived exertion assessed by the Borg scale remained comparable between modalities. This dissociation suggests that treadmill exercise may enable higher absolute physiological stress without a proportional increase in subjective effort, which may be relevant in functional capacity assessment. Similar observations were reported by Gerlach et al. [22], who, in a meta-analysis of studies conducted among patients undergoing cardiac rehabilitation, demonstrated greater improvement in functional capacity during treadmill-based training compared to training on a bicycle ergometer. Mazaheri et al. [6] also demonstrated that during exercise tests performed on a treadmill, patients achieve significantly higher VO_2_peak values than during tests on a cycle ergometer.

The absence of angina during exercise testing is likely attributable to the stable clinical status of the cohort. All patients were clinically stable following myocardial infarction, had undergone successful percutaneous coronary intervention (PCI), and were referred for phase II cardiac rehabilitation after comprehensive cardiological evaluation. Moreover, the submaximal nature of the protocol (85% of predicted maximal heart rate) further enhanced procedural safety.

Taken together, these findings suggest that both modalities allow for safe and clinically useful assessment of exercise capacity, although they may differ in the type of physiological stress they impose and the mechanisms leading to test termination.

This study has several limitations. Its retrospective design limited control over potential confounders such as habitual physical activity, baseline fitness, and detailed functional capacity assessment. The absence of randomization introduces a risk of selection bias, as assignment to treadmill or cycle ergometer testing was based on physician judgment, considering clinical stability, safety considerations, musculoskeletal limitations, and equipment availability.

In addition, exercise capacity was estimated using predictive equations for MET rather than direct cardiopulmonary exercise testing (CPET). The use of different formulas for treadmill and cycle ergometer protocols may have influenced inter-group comparability.

A limitation of the present study is the lack of detailed information on individual medication classes. Therefore, analyses according to specific pharmacological therapies could not be performed, and the total number of prescribed medications was used only as a surrogate marker of overall clinical burden and multimorbidity. Because medications such as β-blockers or other rate-limiting agents may influence chronotropic response and exercise tolerance, their potential impact on the reasons for exercise test termination could not be evaluated. Future prospective studies should include detailed pharmacological data to clarify these relationships.

Despite these limitations, the study reflects routine clinical practice in a real-world cardiac rehabilitation setting, enhancing its external validity and applicability to everyday decision-making.

Importantly, the allocation of exercise modality was driven by standard clinical workflow rather than a predefined selection algorithm, which further increases the pragmatic value of the findings.

Because of the observational design and the limited performance of the regression models, these results should be regarded as hypothesis-generating and require confirmation in prospective studies.

In conclusion, both treadmill and cycle ergometer testing appear to be safe and feasible modalities for assessing exercise capacity in post-myocardial infarction patients. Lower limb pain observed during cycle ergometer testing appears to be primarily related to patient clinical characteristics rather than the modality itself. From a clinical standpoint, this symptom may reflect reduced functional capacity and higher comorbidity burden, whereas treadmill testing may provide a complementary assessment under more global and weight-bearing physiological conditions. Accordingly, the choice of exercise modality should be individualized based on clinical status, functional capacity, and rehabilitation objectives.

## 5. Conclusions

In summary, the most common reasons for terminating exercise tests, regardless of the device used, were patient fatigue and reaching the predetermined heart rate limit, supporting the safety of submaximal exercise testing in this population. Although lower limb pain was more frequently reported during cycle ergometer testing in the unadjusted analysis, this association was not maintained after adjustment for clinical variables in the multivariable model. Given the observational design and the limited explanatory power of the regression models, these findings should be interpreted with caution. They suggest that the observed differences may be related to differences in the baseline clinical profile, including exercise capacity (MET) and comorbidity burden, rather than to the exercise device itself. Therefore, the choice of exercise testing modality should be individualized according to the patient’s clinical status, functional capacity, and rehabilitation goals rather than based on a routine preference for a particular type of equipment.

## Figures and Tables

**Table 1:** Characteristics of the study group (*N* = 336); *n* (%).

Variable		All Patients			Women			Men		
		Treadmill	Bicycle	*p*-Value	Treadmill	Bicycle	*p*-Value	Treadmill	Bicycle	*p*-Value
Age [years]		60.60 ± 8.90 (38–85)	60.60 ± 8.91 (38–85)	0.998	63.24 ± 6.93 (49–76)	63.22 ± 6.93 (49–76)	0.966	59.85 ± 9.27 (38–85)	59.86 ± 9.29 (38–85)	0.995
Body mass [kg]		84.18 ± 15.71 (49–135)	81.72 ± 16.70 (51–140)	0.083	68.10 ± 11.98 (49–95)	65.32 ± 9.81 (51–90)	0.325	88.72 ± 13.52 (66–135)	86.36 ± 15.27 61–140)	0.108
BMI [kg/m^2^]		28.58 ± 3.95 (19.53–44.08)	27.56 ± 4.15 (18.96–45.20)	0.012	26.99 ± 4.30 (19.53–38.29)	25.52 ± 3.22 (18.96–32.87)	0.178	29.03 ± 3.74 (21.39–44.08)	28.13 ± 4.22 (21.38–45.20)	0.039
Cause of Hospitalization	NSTEMI	80 (47.6)	80 (47.6)	1.00	18 (48.6)	14 (37.8)	0.350	69 (52.7)	66 (50.4)	0.621
	STEMI	88 (52.4)	88 (52.4)		19 (51.4)	23 (62.2)		62 (47.3)	65 (49.6)	
EF [%]		52.24 ± 8.33 (24–71)	49.29 ± 8.78 (20–71)	0.006	51.83 ± 7.91 (38–71)	51.69 ± 8.12 (37–64)	0.817	52.35 ± 8.48 (24–70)	48.62 ± 8.87 (20–71)	0.001
Time since cardiovascular event [days]		30.74 ± 20.29 (7–121)	33.65 ± 24.15 (2–154)	0.378	38.11 ± 24.73 (7–121)	27.19 ± 19.20 (7–96)	0.016	28.66 ± 18.43 (7–121)	35.44 ± 25.12 (2–154)	0.028
Number of comorbidities [*n*]		2.80 ± 1.51 (0–9)	3.33 ± 1.42 (0–7)	0.000	2.89 ± 1.58 (0–8)	3.38 ± 1.50 (1–7)	0.130	2.78 ± 1.50 (0–9)	3.31 ± 1.40 (0–7)	0.001
Number of medications taken [*n*]		7.47 ± 1.82 (3–13)	8.05 ± 2.10 (4–17)	0.011	7.78 ± 1.96 (4–13)	8.03 ± 1.95 (4–13)	0.567	7.38 ± 1.78 (3–13)	8.05 ± 2.15 (5–17)	0.010
Smoking current [*n*/%]		59 (35.12)	70 (41.67)	0.217	15 (40.54)	15 (40.54)	1.00	44 (33.59)	55 (41.99)	0.161

BMI—body mass index; EF—ejection fraction; NSTEMI—non—ST—segment elevation myocardial infraction; STEMI—ST— segment elevation myocardial infraction.

**Table 2:** Characteristics of selected exercise test parameters; $\overset{¯}{x}$ ± SD; (min–max) or *n* (%).

Variable	All Patients			Women			Men		
	Treadmill	Bicycle	*p*-Value	Treadmill	Bicycle	*p*-Value	Treadmill	Bicycle	*p*-Value
Borg scale [pts]	14.42 ± 1.95 (8–18)	14.37 ± 1.94 (8–19)	0.779	15.09 ± 1.58 (10–18)	14.65 ± 1.52 (10–17)	0.280	14.24 ± 2.00 (8–18)	14.30 ± 2.04 (8–19)	0.841
MET	7.19 ± 1.70 (3–13.6)	5.28 ± 1.09 (2.7–8.1))	<0.001	6.05 ± 1.18 (3–8.7)	5.36 ± 0.74 (3.4–7.0)	0.002	7.51 ± 1.70 (3.2–13.6)	5.26 ± 1.18 (2.7–8.1)	<0.001
HR rest [bpm]	78.02 ± 10.79 (52–129)	72.46 ± 10.9 (50–113)	<0.001	80.30 ± 9.12 (61–99)	67.84 ± 7.37 (53–84)	<0.001	77.38 ± 11.16 (52–129)	72.46 ± 10.9 (50–113)	<0.001
HR peak [bpm]	128.76 ± 27.05 (84–265)	120.82 ± 20.67 (70–246)	0.004	130.27 ± 25.85 (86–211)	120.54 ± 18.27 (93–197)	0.056	128.33 ± 27.46 (84–265)	120.82 ± 20.67 (70–246)	0.027
HRR [bpm]	50.74 ± 25.84 (7–191)	48.36 ± 20.80 (14–171)	0.549	49.97 ± 24.63 (7–120)	52.70 ± 19.70 (17–140)	0.355	50.96 ± 26.26 (1–191)	48.35 ± 20.80 (14–171)	0.928
SBP rest [mmHg]	119.87 ± 12.18 (90–140)	114.29 ± 14.69 (85–154)	<0.001	119.84 ± 14.65 (90–140)	112.78 ± 14.72 (85–161)	0.013	119.88 ± 11.46 (90–140)	114.29 ± 14.69 (85–154)	<0.001
DBP rest [mmHg]	75.43 ± 7.78 (60–95)	70.28 ± 8.62 (50–91)	<0.001	73.76 ± 8.71 (60–90)	69.08 ± 8.59 (54–92)	0.009	75.90 ± 7.47 (60–95)	70.28 ± 8.62 (50–91)	<0.001
SBP peak [mmHg]	165.59 ± 20.57 (100–225)	168.15 ± 25.23 (107–225)	0.744	157.16 ± 22.22 (100–210)	162.54 ± 20.25 (105–220)	0.297	167.96 ± 19.53 (110–225)	168.15 ± 25.23 (107–225)	0.986
DBP peak [mmHg]	81.17 ± 7.12 (60–100)	81.14 ± 12.25 (53–132)	0.552	79.65 ± 5.61 (70–100)	78.84 ± 10.13 (57–94)	0.489	81.59 ± 7.45 (60–100)	81.14 ± 12.25 (53–132)	0.417
DP rest/100	93.63 ± 17.02 (59.95–170.80)	81.45 ± 16.36 (42.5–151.4)	<0.001	96.73 ± 18.89 (61–133.65)	76.68 ± 13.82 (48.45–112.7)	<0.001	92.77 ± 16.43 (59.95–170.8)	82.85 ± 16.81 (42.5–151.42)	<0.001
DP exercise/100	213.70 ± 52.94 (92.4–477)	202.20 ± 48.68 (82.6–546.12)	0.048	205.35 ± 51.68 (103.2–379.8)	196.41 ± 40.83 (101.85–340.81)	0.261	216.04 ± 53.25 (92.4–477)	203.85 ± 50.71 (82.6–546.12)	0.100
DP reserve	2.11 ± 0.49 (1.2–4.1)	2.53 ± 1.66 (1.3–22.1)	<0.001	1.85 ± 0.30 (1.4–2.6)	2.49 ± 0.58 (1.7–3.9)	<0.001	2.17 ± 0.50 (1.2–4.1)	2.54 ± 1.85 (1.3–22.1)	<0.001

MET—metabolic equivalent; HR—heart rate; HRR—heart rate recovery; SBP—systolic blood pressure; DBP—diastolic blood pressure; DP—double product; DP reserve—double product reserve index; pts—points; bpm—beats per minute.

**Table 3:** Analysis of the reasons for discontinuing or completing the exercise test in the study group (*N* = 336); *n* (%).

Reasons for Terminating the Exercise Test	All Patients	Treadmill	Bicycle	*p*-Value
Fatigue [*n*/%]	185 (55.06)	98 (58.33)	87 (51.79)	0.228
HR limit [*n*/%]	125 (37.2)	60 (35.71)	65 (38.69)	0.573
Blood pressure limit [*n*/%]	12 (3.57)	4 (2.38)	8 (4.76)	0.190
Rhythm disturbances [*n*/%]	7 (2.08)	2 (1.19)	5 (2.98)	0.224
ECG abnormalities [*n*/%]	11 (3.27)	8 (4.76)	3 (1.76)	0.109
Lower limb pain [*n*/%]	39 (11.61)	12 (7.14)	27(16.07)	0.011
Other [*n*/%]	11 (3.27)	5 (2.98)	6 (3.57)	0.500

HR—heart rate; ECG—electrocardiogram.

**Table 4:** Analysis of the reasons for interrupting or terminating the exercise test in the study group of women (*N* = 74); *n* (%).

Reasons For Terminating The Exercise Test	All Women	Treadmill	Bicycle	*p*-Value
Fatigue [*n*/%]	38 (51.35)	21 (56.76)	17 (45.95)	0.352
HR limit [*n*/%]	27 (36.49)	14 (37.84)	13 (35.14)	0.809
Blood pressure limit [*n*/%]	0 (0)	0 (0)	0 (0)	-
Rhythm disturbances [*n*/%]	1 (1.35)	0 (0)	1 (2.7)	0.5
ECG abnormalities [*n*/%]	2 (2.7)	1 (2.7)	1 (2.7)	0.753
Lower limb pain [*n*/%]	12 (16.22)	4 (10.81)	8 (21.62)	0.172
Other [*n*/%]	6 (8.11)	3 (8.11)	3 (8.11)	0.663

HR—heart rate; ECG—electrocardiogram.

**Table 5:** Analysis of the causes of interruption or termination of the exercise test in the study group of men (*N* = 262); *n* (%).

Reasons for Terminating the Exercise Test	All Men	Treadmill	Bicycle	*p*-Value
Fatigue [*n*/%]	147 (56.11)	77 (58.78)	70 (53.44)	0.384
HR limit [*n*/%]	98 (37.41)	46 (35.12)	52 (39.7)	0.444
Blood pressure limit [*n*/%]	12 (4.58)	4 (3.05)	8 (6.11)	0.188
Rhythm disturbances [*n*/%]	6 (2.29)	2 (1.53)	4 (3.05)	0.342
ECG abnormalities [*n*/%]	9 (3.44)	7 (5.34)	2 (1.53)	0.086
Lower limb pain [*n*/%]	27 (10.31)	8 (6.11)	19 (14.5)	0.025
Other [*n*/%]	5 (1.91)	2 (1.53)	3 (2.29)	0.5

HR—heart rate; ECG—electrocardiogram.

## Data Availability

The statistical data used to support the presented findings may be obtained by sending a request to the corresponding author due to privacy constraints.

## Abbreviations

BMI	Body Mass Index
CABG	Coronary Artery Bypass Grafting
CPET	Cardiopulmonary Exercise Testing
DBP	Diastolic Blood Pressure
DP	Double Product
DP reserve	Double Product Reserve Index
EAPC	European Association of Preventive Cardiology
ECG	Electrocardiogram/Electrocardiographic
EF/LVEF	Left Ventricular Ejection Fraction
ESC	European Society of Cardiology
ET	Exercise Testing
HFmrEF	Heart Failure with Mildly Reduced Ejection Fraction
HR	Heart Rate
HRpeak	Peak Heart Rate
HRrest	Resting Heart Rate
HRR	Heart Rate Recovery
MET	Metabolic Equivalent
NSTEMI	Non–ST-segment Elevation Myocardial Infarction
PAD	Peripheral Artery Disease
PCI	Percutaneous Coronary Intervention
RAMP	Ramp Protocol (Continuous Incremental Workload Protocol)
SBP	Systolic Blood Pressure
STEMI	ST-segment Elevation Myocardial Infarction
VO_2_max	Maximum Oxygen Uptake
VO_2_peak	Peak Oxygen Uptake
